# Proactive learner empowerment: towards a transformative academic integrity approach for English language learners

**DOI:** 10.1007/s40979-022-00111-2

**Published:** 2022-09-26

**Authors:** Elaine Khoo, Sohee Kang

**Affiliations:** 1 Centre for Teaching and Learning, University of Toronto Scarborough, Toronto, Canada; 2 Department of Mathematics and Computer Science, University of Toronto Scarborough, Toronto, Canada

**Keywords:** Academic integrity, Academic writing, Learner-centred, Post-secondary, Learner empowerment, Transformative learning, COVID-19

## Abstract

Socializing students to Academic Integrity (AI) in the face of great cultural, linguistic and socioeconomic diversity in the student population in higher education calls for innovative strategies that are aligned with equity, diversity and inclusion principles. Through a mixed method of quantitative analysis of learner engagement data from the Learning Management System (LMS) and analysis of anonymous evaluation survey, along with thematic analysis of students’ open-ended responses in the evaluation survey, the authors explored how students responded to AI Socialization during a 4-week non-credit, online co-curricular program called ‘Reading and Writing Excellence’ (RWE). Nine groups of undergraduate students (*N*=182) from 34 disciplines in different global locations during the COVID-19 pandemic were introduced to a curated set of AI online resources. Through a learner-driven, instructor-facilitated approach the AI Socialization also engaged students in language development and empowered them to communicate about their disciplinary course topics through written journal entries, receiving instructor feedback that increased their cultural and linguistic capital for further academic writing. This approach led to a high volume of written output (on average 6064 words per student written over a 4-week period). Nonparametric ANOVA was used to establish that low-proficiency students were able to produce as much written output as their more proficient peers. Survey results for various aspects important to academic integrity show students’ self-perception of readiness for academic writing: paraphrasing and summarizing (92%); organization of ideas (92%); critical thinking (93%); logic/argument (92%). Insights gained about educative engagement, language development and learner empowerment that can help students from diverse backgrounds to avoid Academic Integrity Violations (AIVs) and gain transformative access and success in higher education are incorporated into a set of recommendations that are applicable to a wide range of teaching contexts.

## Introduction

Cultural, linguistic, socioeconomic and educational differences in the diverse student demographic in higher education present challenges for students, faculty and institution when addressing issues of academic integrity. Due to internationalization, immigration, and universal access, some students may encounter serious challenges as their previous experiences and training may be quite different from those valued by their university, leading to a great deal of acculturative stress. International students from Asia, Central/Latin America and Africa were found to have greater acculturative stress than their counterparts from Europe (Yeh & Inose, 2003). Many domestic students from immigrant families (Roessingh & Douglas, 2012), lower socioeconomic status (SES), minority and marginalized groups as well as mature students, whose pathway to university had been unconventional, have similar transition challenges, and need to be adequately supported to help them acculturate to the academic expectations at university. In this paper, English Language Learners (ELLs) will be used to refer to both international and domestic students whose level of Academic English needs improvement so they can cope efficiently with academic demands. Students with low academic English levels have been over-represented in cases of academic integrity violations (Bretag et al., 2019). Much is assumed about the academic skills and metacognitive strategies students entering university should have in order to meet the academic integrity expectations at university. Instead of leaving it to students to bear the consequences of the mismatch of their perception of academic integrity and the institutional expectations of academic integrity, East (2016) emphasizes that universities cannot “assume that all students enrolling in its courses come with experience of local scholarship expectations” (p. 484).

Learning how to do source-based writing to meet the criteria for Anglo Western academic writing is a fundamental skill for all learners in English medium universities, while failure to meet that threshold of skill can lead to repercussions such as being charged with academic integrity violations (AIV). Some ELLs resort to seeking “contract cheating” (Clarke & Lancaster, 2007) providers to write assignments as a result of the ELLs low confidence in their writing skills (Mathrani et al., 2021). In addition, some ELLs struggle with their limited English skills and resort to using paraphrasing tools on the internet (Perkins et al., 2018; Rogerson, 2017).

Undergoing the process for AIV with the university’s AI Committee (AIC) (i.e., a committee that investigates academic misconduct) can be traumatic, reported by students as “the hardest, most challenging or worst experience of their lives” (Pitt et al., 2021, p. 663). Some students described how the intensity of the experience “reverberated through their lives in the weeks and months following the university AIC outcome” (p. 664). The AIC outcome can result in tremendous financial stress (i.e, wanting to secure funds to repeat the course without burdening their families) and feeling stigmatized among their peers. An additional burden is the constant fear of accusation of textual plagiarism due to students’ low level of English proficiency, which may result in learners experiencing a negative change in their relationships to their peers and instructors (Pitt et al., 2021; Tindall et al., 2021). The stigma and traumatic experience of a misconduct outcome have resulted in the need for some institutions to put the affected students on “suicide watch” (Robinson & Openo, 2021, p. 34). The emotional labour involved in policing and following through with allegations have been unwelcome emotional labour for instructors (Hutchings, 2014; Robinson & Openo, 2021).

With AIVs having extensive negative impact on both instructors and students, it is ethical from an equity perspective for institutions to seriously examine ways of supporting these equity-deserving students in proactive ways so they develop the language repertoire, academic literacy skills and competence they need. Otherwise, as noted by (Murphy, 2022), “it borders on unethical to keep taking their tuition dollars and de facto promising they can succeed” (p.85). Developing these skills take time, sustained effort on the part of students and sustained support from institutions. However, there are no studies we are aware of which take a sustained proactive developmental and empowerment approach of a non-remedial nature to support equity-deserving students with respect to academic integrity.

In this paper we will first outline the key challenges that students face with academic integrity issues when coping with source-based writing. Then we will describe the research on a pandemic-induced introduction of academic integrity socialization into a student-driven, instructor-facilitated writing support program that embeds students’ disciplinary writing needs. Next, we will share the pedagogical insights that lead to recommendations for integrating AI socialization into many teaching contexts.

## Literature review

### Challenges students face regarding AI issues

For many students, the higher education environment may seem like an “alien environment” (Askham, 2008) where they encounter unfamiliar expectations of participation and contribution (Heng, 2017, 2018). During university orientation, it is likely that many students feel overwhelmed and lost when informed about an institution’s academic integrity policy and penalties for breaches, and they “live with the fear that if they were caught plagiarising, they might be accused of something close to ‘criminal behaviour’ and punished” (Hutchings, 2014, p. 313). The multiple challenges students face with AI can be broadly categorized as (a) cultural and education experiences before university; (b) need to learn about the academic culture of enquiry and evidence-based arguments; (c) linguistic repertoire for Academic English; (d) temptation of seemingly easier ways to get their assignments done (Mathrani et al., 2021; Yorke et al., 2020). As cultures vary greatly in what is considered knowledge, and educational systems vary in how students’ academic performance is measured, East (2016) cautions that universities cannot assume that students will arrive with a clear understanding of academic integrity. A Canadian study found a mismatch between faculty expectations that students arrive knowing how to write and the reality that students arrive expecting to be trained on how to write (Peters & Cadieux, 2019). Our study underscores the importance of actively socializing students to the expectations of AI, and facilitating their ability to develop the necessary skills for practicing AI.

### Addressing key issues

Resolving students’ challenges with AI requires a pedagogical approach that addresses what students say they need—more information and support to develop good academic writing skills without fear of sanctions (Adam, 2015). It is necessary to have a pedagogical approach that demystifies academic culture so students understand the value of citation and referencing practices in presenting an evidence-based scholarly work. This pedagogical approach needs to help students (a) draw upon previous cultural and education experience to realize where differences in definitions, expectations, perceptions and practices about AI might exist; (b) embrace emerging junior scholar identity where they can be producers (not only consumers) of knowledge and present evidence-based arguments; (c) exponentially expand vocabulary and writing skills for academic writing; (d) feel well-scaffolded in developing the mindset and skills that enable students to exercise agency in writing assignments rather than resort to committing AIVs.

Educators need to take a more inclusive and critical approach to help students learn academic writing at the university and related ethics (Eaton, 2022; Kim & Uysal, 2021). With increasing acknowledgement of the social injustice of positioning students who are from different cultures as perpetrators of AIVs, scholars have advocated for a *pedagogical approach*. Some (i.e., Leask, 2006) argue for a change from the colonial mindset of negatively stereotyping learners from culturally ‘other’ backgrounds as inferior, and instead strive to develop pedagogy that recognizes the value of difference and encourage learner individuality. Bretag (2013) advocates for educational institutions to move away from the prevalent focus on deterrence, policing, and punishment towards fostering a positive culture of academic integrity. In a similar manner, when students are being investigated for AIV, institutions take a developmental approach by following the four steps of assessment, assignment, education and evaluation where “the assessment should not be done *to* the student but *with* the students (emphasis by original authors) (Bertram Gallant & Stephens, 2020, p. 62). Recognizing that “Eurocentric middle-class views” (Eaton & Burns, 2018, p. 345) are privileged within Western institutions and students unfamiliar with the expectations for writer-responsible writing convention are mainly left to their own devices to cope with avoiding AIVs, Eaton and Burns advocate for a culturally responsive pedagogy that is “inclusive, dynamic, supportive, while still maintaining academic rigor” (p. 343). Additionally, Bretag et al. (2019) highlight a conspicuous gap in the field, noting that “despite two decades of research which has pointed to the need to direct resources toward more systematic approaches to students’ language and learning development, little progress appears to have been made”(p. 1850). Eaton (2022) emphasizes the importance of recognizing the importance of “decolonizing academic integrity in practice, policy and research” (p. 10) instead of perpetuating “colonial norms” (p.8). To begin taking a step towards this decolonization, institutions need to examine if the current levels of support for these groups is adequately transforming their learning experiences or continuing to subjugate these groups through placing unreasonable expectations of academic integrity on them before giving them a chance to learn how they can assert themselves and bring their lived experiences into their new learning environment.

### Language challenges that ELLs face with source-based writing

Many ELLs might not have the breadth and depth of linguistic repertoire to cope with academic reading and source-based writing. Gullifer & Tyson (2010) note that “good academic writing is contingent on developing sound skills in both research and writing, critically reading and comprehending appropriate sources, careful note-taking, paraphrasing, judicious use of quotations and giving credit to authors for their ideas and writing” (p. 464). Source-based writing is more demanding than most prior writing that ELLs have had to do. Limited vocabulary makes critical reading a great challenge since students need to know at least 98% of the words on the page to comprehend the text (Nation, 2006) and for academic texts, this translates into the need for 10,000 word families (Khoo & Kang, 2017). Writing in their weaker language that involves skilful sentence construction, utilizing precise vocabulary and grammar (Chan, 2010), identifying and evaluating salient information in source text to summarize or paraphrase in newly composed text (Shi, 2004; Wette, 2010) is demanding. What is more linguistically challenging than summarizing and paraphrasing is the ability to quote as it “involves incorporating someone else’s wording into one’s own text in such a way that the interface is coherent and fluent” (Pecorari, 2016, p. 543). Due to the complexities of academic writing, ELL writers were found to incorporate longer chunks from source texts into their writing compared to their more fluent peers (Keck, 2006, 2014). However, scholars have pointed out that incorporating source text is a necessary part of the process of learning to write in the academic genre and to acquire experience with citation practices (Chandrasoma et al., 2004; Howard, 1995). Students need to have practice using academic English to the point that they are fluent enough to write their assignments. That said, opportunities to practice are limited. In Graves et al. (2010)’s survey of course syllabi, they found that “students were rarely asked to submit work that was not subsequently graded” (p. 302). ELLs have been stereotyped negatively as likely plagiarists (Heng, 2018; Moosavi, 2021) and pointedly excluded by peers in group writing assignments, causing them to feel hurt (Guo & Guo, 2017). As a result, creating the safe conditions for academic language usage and scaffolding academic integrity can counter this incorrect negative stereotype.

### Special concern about remote learning during the pandemic

The sudden pivot to emergency remote teaching and learning resulted in many instructors significantly increasing the volume of written work required of students (Motz et al., 2021). For ELLs, this may increase their burden exponentially. It is unreasonable to expect these students to cope with the increased number of writing assignments, develop logical arguments based on Western norms which may be culturally different from students’ native culture and writing conventions, and summarize and paraphrase without “intertextual missteps” (Jamieson & Howard, 2019, p. 78) that can lead to their being accused of plagiarism. Many students living in their home countries had few opportunities to use English in their daily interactions. Thus, providing students with the relational opportunity to use English to communicate their ideas substantively with a supportive instructor, facilitating their AI socialization and academic language skills development was an easily overlooked necessity during the pandemic. From an equity standpoint where ELLs and equity-deserving students need support, it is worth examining opportunities for ELLs and other equity-deserving students to communicate meaningfully on substantive course materials so that they are not vulnerable to accusations of AIVs.

### Learner self-regulation and empowerment

Students unfamiliar with Eurocentric academic integrity practices and disadvantaged by the lack of Academic English proficiency may be frustrated not only by the lack of support but also seeming exclusion by instructors and peers as they are perceived as Other (Guo & Guo, 2017). Other scholars (e.g. Mott-Smith, 2013; Murphy, 2022) noted that academic integrity is usually presented in negative terms in the form of prohibitive warnings and consequences for violations, resulting in students paralyzed with anxiety and fear. Compassionate proactive actionable guidance is needed to enable students to cite sources, and learn that the primary purpose of paraphrasing is to enable the writer to have the power to recast the original writer’s words in a manner that serves the student writer’s authorial purpose (Shi et al., 2018), rather than mere linguistic manipulations in order to avoid plagiarism (Rossi, 2022). In order for students to develop competence with writing in Academic English and learn that they are expected to exercise agency in their writing, it is important to provide a safe space to practice language skills and academic integrity. At the university level where students are expected to be self-regulated learners (Zimmerman, 2002), it is necessary to promote learner agency, which (Martin, 2004) defines as “the capability of individual human beings to make choices and to act on these choices in ways that make a difference in their lives” (p. 135). For students to develop academic integrity values through meaningful writing, it is necessary to leverage learner agency by engaging students in decision making about their learning and language practice, as well as by familiarizing themselves with the value of using citations as part of their emergent scholar identity that synthesizes literature and generates knowledge.

## Context of study

When the global COVID-19 pandemic caused universities to pivot to remote teaching, there was concern that ELLs’ learning challenges previously mentioned were exacerbated. Equity-deserving students whose circumstances precluded their gaining familiarity with Academic English and academic writing conventions also needed support during these challenging times. An expedient way to respond to students’ needs was to re-envision a long-running co-curricular program, Reading and Writing Excellence (RWE), that had been supporting students in developing their academic writing skills in a learner-driven, instructor-facilitated model to fully online, and incorporate an AI socialization component. AI socialization is defined as the

process in which students are welcomed into a safe and supportive space (virtual and/or physical) to explore the AI expectations of their institution which are presented in a comprehensive, learner-centred manner. Students have the opportunity for questioning, clarifying with a supportive person, and reflecting about AI in ways that are personally meaningful in relation to their previous socio-cultural and educational training. As a further step, the learner has agency to practice aspects of AI, along with language development, as they incrementally establish their new learner identity in the academic community without the stress of being graded. Thus, AI socialization occurs when a student learns how to engage in scholarly conversations using sources and references in a way that becomes organic to their identity as a junior scholar, rather than perceive AI as a punitive measure to be feared.

This paper aims to examine to what extent students are willing to engage in AI socialization and develop their critical academic reading and writing. We examine the impact of this model of support that emphasizes learner agency in the process of students learning how to bring themselves into the academic learning space without denying their identity and existing knowledge, but rather through exploring their new space, making intellectual connections, and learning how to assert their own perspectives while developing their academic voice. Research ethics approval was granted to study this pedagogical approach retrospectively, making secondary use of data.

The research questions in this study are:

- RQ1: How much voluntary writing were students willing to do in this program?
- RQ2:What were students’ perceptions of whether the program had helped them improve their academic writing and critical thinking skills?
- RQ3: Did students perceive the program to have a transformative impact on them?

## Methods

### Participants

Participants came from a convenience sample of students in the RWE program conducted by the Centre for Teaching and Learning. Among the 182 students in the program from 34 disciplinary programs, 97 were in their first year of study, 34 were in their second year of study, 31 were in their third year of study, 18 were in their fourth year of study, and two were in their fifth year or above. Sixty-six students self-declared as international students while 116 were domestic students. Seventy-four students self-reported to speak English as a first language while 108 self-reported that English was not their first language. Since this analysis was based on secondary use of data that was kept for program administration, no information on gender and age of the students were available.

### Materials and procedure

#### Establishing level of starting Academic English level

Students’ starting Academic English level was indicated through a Diagnostic English Language Needs Assessment (DELNA) score. The DELNA Screening is a form of post-entry language assessment (PELA) that is used in Australia and New Zealand as a means of efficiently identifying “students who would benefit from additional language support at an early stage in their study”(Elder & Read, 2015, p. 26). Based on the DELNA Screening, students categorized as Band 1 would be those most linguistically at risk (Elder & von Randow, 2008). Band 3 students are generally not considered linguistically at risk based on their vocabulary and ability to read. Band 2 students are in a grey area. The priority for providing spots for students in this free co-curricular language program is in the order of first, Band 1, then Band 2 and finally Band 3 students.

#### Engagement in the support program

Students begin by going through the orientation webpages on the Canvas learning management systems (LMS) and then logging into the Discussion board that been set up as a dedicated thread between each student and the instructor. An instructor may be assigned many students, but a student is assigned to only one instructor. On Day 1, students listen to a 2-minute video of their assigned instructor’s welcome message on the ‘Meet Your Instructor’ page, and in response write a self-introduction to enable the instructor to understand each student’s background, aspirations, anxieties and needs. On Day 2, the student responds to a Journal entry #2 prompt designed to invite learner agency in the process of finding out about AI in the institution, and the gap between what they know about AI and AI expectations of the institution. This website communicated AI as a means to help students self-regulate to achieve their goals. By presenting these resources as hyperlinked webpages it encouraged students to follow up on links that they find meaningful to them. This allowed each student to follow different paths in the exploration of AI. Students who were completing the program for the first time respond to the following prompt:

**Check out these links and identify some things that you learned that you didn't know about before. Also comment on how your investment in yourself through this program relates to academic integrity.**

The links provided were (a) a student-friendly librarian-curated website hosted on the LMS that explains AI in non-threatening terms (i.e., in positive tones), with links to two encouraging videos featuring faculty inspiring students to see the value of practicing academic integrity as part of their personal and professional development; (b) Understanding and Avoiding Plagiarism (U of G Library, 2014) and (c) Avoiding plagiarism (Excelsior online writing lab, n.d.)

Returning students who had previously completed the program could respond to the following prompt:

**Since one of the goals of the program is to help students develop their voice and express their thoughts, perhaps you might like to share what you think might be innovative ways to help students practice academic integrity and avoid plagiarism.**

**However, if you prefer to re-look at the 3 links in the previous section and share some thoughts about them that would be useful for faculty members to consider, please feel free to do so**.

The importance of having competence in reading/writing in Academic English is generally overlooked when imposing academic integrity policies on students. This is addressed in the RWE program by creating the opportunity for students to practice:

Students read their choice of course readings for 40 minutes each day and wrote a journal entry for 20 minutes each day. From Journal Entry #3 onwards, each journal entry was recommended to have a summary component where the learner summarized the texts they had read and then offer their own perspective. Each journal entry was expected to be at least 250 words, which was a reasonable word fluency goal to set for 20 minutes of writing. Students who are not able to reach 250 words initially were encouraged to write what they could in order to develop their skills. Instructors responded 2-3 times per week to students’ ideas in a friendly and supportive manner to encourage students’ motivation to write. Students met their instructor virtually for 30 minutes every two weeks. In this program, students exercise learner agency in choosing the text to read and what to reflect on in their journal entry, and instructors facilitate development by responding according to the unique needs of individual students.

Since students are writing to the specific instructor assigned to support them, every journal entry is at the level of proficiency that the individual student is capable of writing at that time. The journals are not graded, and instructor feedback aims to help students to develop critical thinking about their topics and motivate them to practice AI when writing. Instructors provide supportive, motivating feedback that helps students feel they belong, and enjoy expressing their thoughts and relating the new information about AI and their course topics to their own lived experience and culture.

#### Engagement Data from Learning Management System

The LMS is a rich source of learning engagement data, and objective measures of learner activity on the LMS have been “positively associated with engagement and achievement” (Motz et al., 2021, p. 71). For this analysis we extracted a subset of the engagement data i.e., that pertaining to word count of students’ journal entries. Word count has been established as one of the measures of fluency (Crossley et al., 2013; De Angelis & Jessner, 2012; González, 2017). Since the number of words students produce in this program for one month is the most direct and objective data about students’ language usage, the total number of words written by each student for each journal entry was compiled from the anonymized data set that was downloaded for course evaluation purposes and the production of internal reports. Since students were writing about different readings daily, student’s written output represents students’ practice at using language necessary to communicate to others about topics in their respective disciplines, thus equipping them to be able to work on their upcoming assignments instead of seeking help that may constitute AIV.

#### Anonymous evaluation form

At the end of the one-month program, the program administrator sent students a link to an anonymous survey form to provide feedback for program improvement. Students were anonymously queried through 3 question formats: (a) forced binary (b) multi-category Likert scale and (c) open-ended. Given that the program was voluntary, and students had pressing end-of-semester deadlines and survey completion was not mandated, there was a need to ensure speed and efficiency in reliable data collection. For the eight survey items aimed at determining the academic writing and critical thinking skills aspects of the intervention, the forced binary format was used, as previous research (Dolnicar et al., 2011; Grassi et al., 2007) showed that it enabled participants to respond more quickly without compromising the validity of the data. Meanwhile, for the 11 survey items aimed at eliciting students’ perceptions of transformative impact along different dimensions of their learning experience, the Likert-scale format was used as it was better able to capture the “intermediate shades of respondent opinion” (Dolnicar et al., 2011, p. 247). End-of-program surveys were anonymized in a Microsoft Excel file prior to analysis.

#### Data analysis

The anonymized data was subjected to statistical analysis by the second author in programming language R. Baseline student characteristics were summarized as number and proportion, as all baseline characteristics were categorical variables. Word count distribution was visualized by histograms for the total group and as bar plots by Academic English Band. Differences between instructor groups was assessed using non-parametric Analysis of Variance (ANOVA) test, Kruskal-Wallis ranksum tests. Results with two-tailed p-values smaller than α=.05 were considered statistically significant. On the end-of-program survey, questions about perceived skill improvement, which were binary in nature, were expressed as number with proportion, while questions about perceived transformative impact, which were Likert in nature, were expressed as mean with standard deviation. Finally, open-ended responses were qualitatively analyzed manually by the first author through the identification of themes and subsequently student comments were coded to those categories, and analyzed through thematic analysis (Braun & Clarke, 2012).

For RQ1, we analyzed the download of the LMS engagement data and compiled the summation of word count of students’ journal entries.

For RQ 2, we analyzed the forced binary questions in the anonymous end-of-program survey. This cluster of questions to survey students’ perception of support were of two categories i.e., for developing (a) academic writing skills and (b) critical thinking skills

For RQ3, we analyzed the quantitative data from the 11 questions that students responded to on a 5-point Likert scale in the anonymous end-of-program survey. We also analyzed qualitatively the comments that students made to the two questions: What did you find to be the most helpful part of the program? What would you like to see changed in the program?

## Results

### Nature of journal writing task

It is made clear to students that their journal writing is risk-free and does not require editing and revision. The unedited sample from Student HW018 shown below illustrates what an ELL reflected upon responding to the AI prompt shown on page 8.

Academic integrity that shows someone’s effort and honesty are important in study successfully at university. Academic dishonesty will lead to serious consequences. So, we need to take it seriously. There is a wide range of violations of academic integrity, but the most noteworthy one is plagiarism. Except for some dishonest reasons, some students do not mean to but violate academic integrity. How do we avoid these? In my opinion, it can be avoided to some extent by improving reading and writing skills.

First, some students may have no inspiration when writing, they may have been inspired by reading among others, but they cannot distinguish which part is theirs and which belongs to others. As a student who is not good at writing, I will also have the same dilemma. RWE will help me get more inspiration through encouragement and guidance. Meanwhile, teach me to distinguish between these thoughts and clear my mind.

Second, sometimes students use what others say to prove their point of view, but have no clear understanding of plagiarism, they may constitute academic plagiarism by not quoting the correct format. RWE can offer us to establish a correct mode of writing, point out problems in our writing which we may not notice before and might constitute a breach of academic integrity.

Finally, critical thinking is important, it not only helps us avoid plagiarism because we do not always agree with others and use others opinions which is easy to cause plagiarism, but also helps improve the quality of writing, and further help with academic success. I know that I am lack critical thinking, in the RWE program I will learn by reading and writing more and then edit my writing to develop my thoughts.

The following unedited sample from student BW001 illustrates the student articulating the gut response to an article read, and the “raw” nature of the writing with fragments and run-on sentences, incomplete ideas, etc. This is valuable work as it engages students in thinking about course material read and capturing thoughts about it in writing. This can be likened to prewriting for an assignment. Notable is that the student is getting practice communicating their thoughts to the instructor.

In the article "the Purpose of Education", Jr. (2012) talks about the object of education. Education can lead us to view the “legions of half-truth, prejudices, and propaganda” from a higher perspective (Jr., p. 5). As we are in society, we should confront these situations at all times. People cannot discern right or wrong if they are not been well-educated.

It is time for the educated people not to be overwhelmed by the rush of the prejudiced ideologies, such as anti-the Black, anti-Muslim, and anti-homosexuality. However, the well-educated people could be invaded by the presses, corps, even the pulpits. We all know that thinking intensively is not easy to develop, it should unite all the educators and students in the information-surrounded world. That is the object of education—making people sharply view the world. For example, when people are flooded with various kinds of information, they should keep it at a distance, to look for the true meaning or the purpose of the information other than taking in all the information regardless of whether it is true or prejudiced. Stepping at a higher stance to view the legions of propagandas and the half-fake.

When the instructor responds to a student, the instructor aims to build a relationship of trust that motivates the student to continue to read and write throughout the month. The writing practice is essential to enable students to build their linguistic repertoire for critical writing and practicing academic integrity. As such, instructor responses are encouraging and specific in addressing students’ ideas in their journals. This quick turnaround is much valued by students as they can learn from the instructor response to improve subsequent journal entries. In this AI socialization process the instructor uses a cordial and friendly tone as illustrated below.

Thank you for your thoughtful journal entry.

This is just a gentle reminder that when you are citing an author, you want to include their entire last name. In this instance, it looks like you're only citing "Jr." which is an abbreviation for "Junior," a word that is typically used at the end of a last name to show that he or she is the son or daughter of the person of the same name. In this instance, you would want to refer to the author as King Jr.

Your journal touches upon many aspects relating to the purpose of education. It might be helpful if you were to consider the context of Martin Luther King Jr.'s essay and his position as a person of colour discussing education in 1947. Do you agree or disagree that some of the ideas he discusses are still relevant today? Why or why not?

I think your point that people need to be critical and be able to evaluate information effectively is a good one and I would encourage you to connect these ideas further by using King Jr.'s essay to help support your position. Doing so, demonstrates your understanding of the text and strengthens your argument. In other words, do you think King Jr. would agree with your position?

Keep up the excellent work!

### Volume of voluntary writing

#### RQ1: How much voluntary writing were students willing to do in this program?

**Fig. 1:**
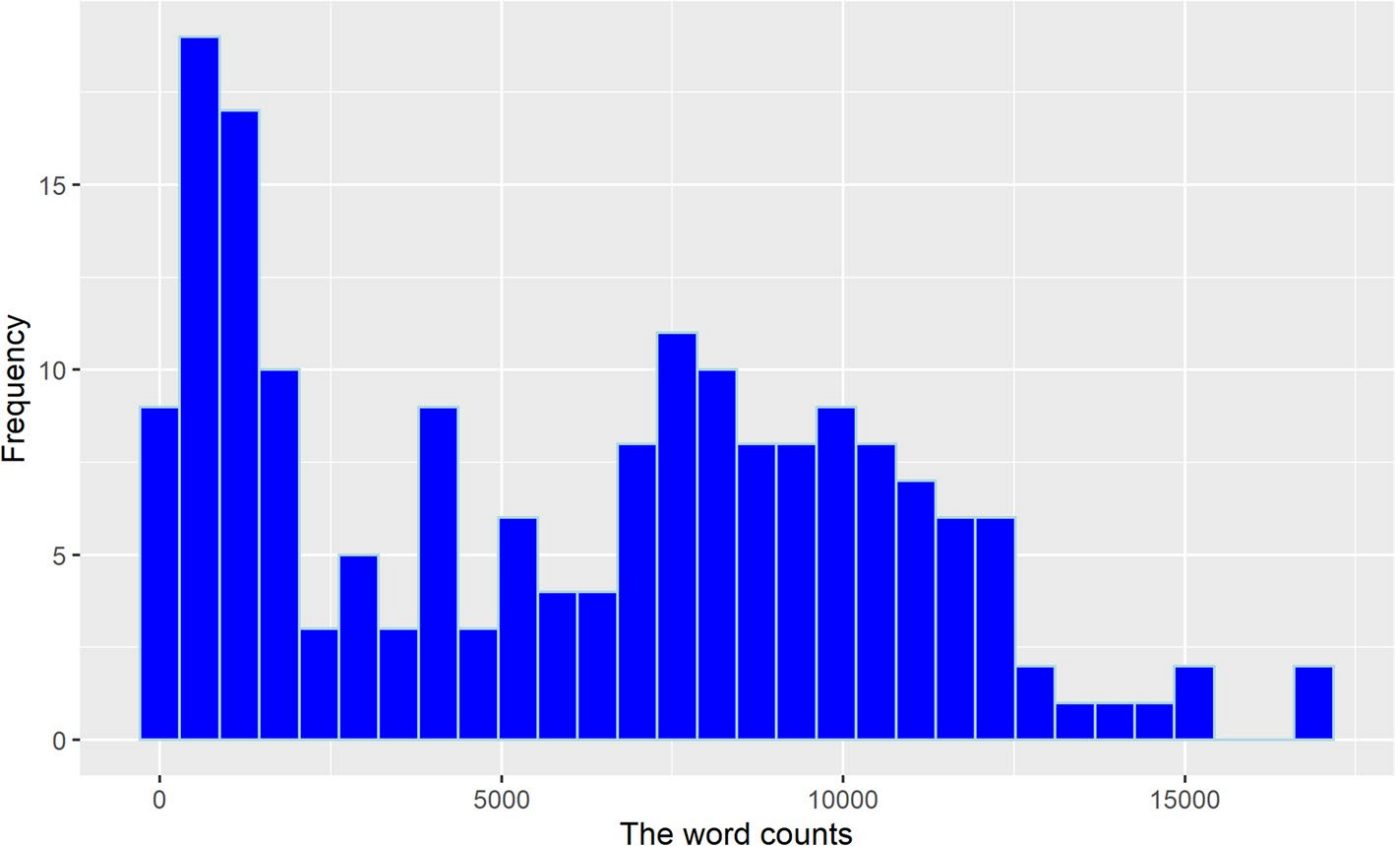
Distribution of total journal word count journals in one month and frequency of the counts

Across the 9 groups of 182 students, the general trend is a high volume of voluntary written output within the one month (Fig. 1). The average word count produced was 6064 and the maximum number is 17038. The standard deviation is 4353, indicating a wide variation in output. As seen in Fig. 1, although the majority of the students wrote more than 5000 words in one month, a small number did not write at all or wrote less than a total of 1000 words.

Analysis of the word counts of students in the different bands (Table 1) showed that although Band 3 students seemed to have written the most and Band 2 the least, there was no statistical difference based on the nonparametric ANOVA (Analysis of Variance) test, Kruskal-Wallis rank sum test (*p* = 0.3242) among the students in all three bands with respect to the average total word count of each individual student.

**Table 1:** Summary table of volume of written output among students of different proficiency levels

Band	*N*	*M*	*SD*
1	86	5844	4491
2	30	5374	4186
3	66	6664	4232

**Fig. 2:**
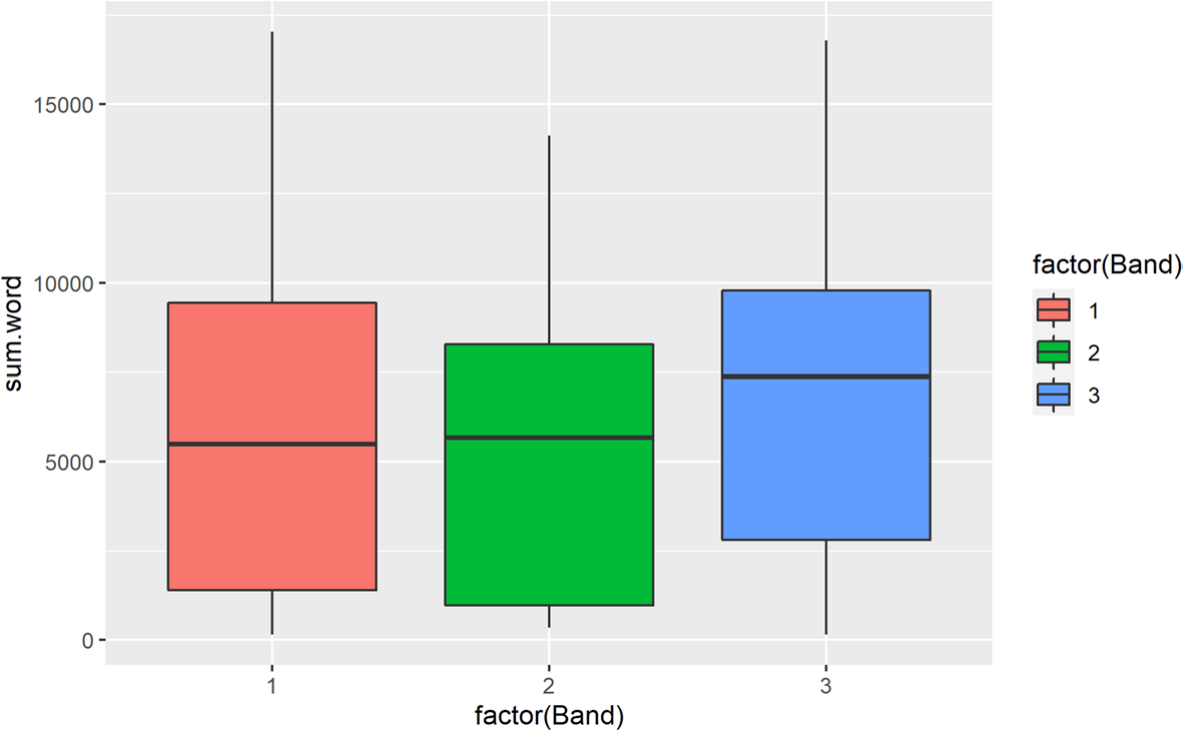
Average total wordcount distribution in the three bands of Academic English ability

Although Band 1 students, based on their low level of Academic English proficiency, would have been expected to produce the least amount of output, they were in fact motivated to write a comparable amount to Band 3 students. In fact, the whisker plot (Fig. 2) indicates that the outliers in the upper bound (greater number of total words) in written output in Band 1 and Band 3 are similar.

##### Students’ perception of what is learned through program

The anonymous survey had a high participation rate of 130 out of 182, which is 71.4%, so we can be confident that the results are representative of the student experience. A 36% response rate to survey questionnaire was considered acceptable (Love & Smith, 2003)

### RQ2:What are students’ perceptions of whether the program had helped them improve their academic writing and critical thinking skills?

A high percentage of students responded with “Yes” to items in the survey related to the key strategies in academic writing and critical thinking: paraphrasing and summarizing skills (92%); organization of ideas (92%); critical thinking about disciplinary topics (93%); logic and argument structure (92%); awareness of academic audience (84%).

A lower percentage of students responded with “Yes” to items related to language usage development and grammar (78%), academic vocabulary and formal writing style for university assignments (76%). The lowest percentage of “Yes” was given to “Improve my in-text citation style” (54%).

The Kruskal Wallis test, a non-parametric ANOVA test, was used to see if students in different groups had different perceptions. According to the Kruskal Wallis test results, there is no statistical difference between the groups assigned to students for survey questions related to academic writing skills, but there is a significant group difference for survey items related to critical thinking skills (Table 2).

**Table 2:** Students' perception of areas of learning

Cluster description	*M*	*SD*	*χ*^2^	*p*
Cluster 1: Four survey items about academic writing skills	4.22	1.2	11.885	.1564
Cluster 2: Three survey items about critical thinking skills	2.39	0.7	17.751	.02317 *

*p < .05

The perception scores of two clusters are high, and there is no statistical difference for academic writing skills scores by each tutor, however, there is a significant tutor effect for the critical thinking skills scores.

### Students’ Perception of Impact

#### RQ3: Did students perceive the program to have a transformative impact on them?

Students’ perceptions of the impact of participation were measured through four clusters of items that students respond to on a 5-point Likert-scale in the anonymous survey where 1=Strongly Disagree and 5=Strongly Agree. The overall average score of 11 questions is 4.0 out of 5 (Table 3).

**Table 3:** Items on the anonymous survey that students respond on 5-point Likert scale

Cluster A (Perception of own cognitive and writing ability)	• I am more confident to write about topics in my courses • I have the knowledge and skills to ensure I have academic integrity in all my writing • I understand my course content better because I am writing about them in RWE • I have expanded my academic language ability through reading and writing in RWE
Cluster B (Perception of value of feedback)	• Every response from my instructor provides new information that improves my writing ability • My instructor’s responses were specific to my individual learning needs and language level so I developed my skills quickly in one month.
Cluster C (Perception of relationship with instructor)	• I have a positive relationship with my instructor so I enjoy sharing my thoughts with him/her and get feedback • My instructor’s encouragement and support help me overcome my anxiety/fear about academic writing
Cluster D (Perception of self-regulation impact)	• I improved my time management skills because of having to write every day in RWE • Due to doing RWE, I am keeping up with reading my course materials each week. • Without RWE, I would not have written so much in one month.

Since students were assigned to 9 groups, each with a different instructor, we examined the four clusters of the survey questions to see whether there was any statistical difference in the responses by the groups that students were assigned to.

The non-parametric ANOVA test verifies that for each cluster there is no statistical difference in the mean of clusters among the groups. We can conclude that regardless of the group that students were assigned, their perception of improvement were high based on the mean scores. They are all above 4 on a 5-point Likert scale (Table 4).

**Table 4:** Non-parametric ANOVA, Kruskal Wallis test results

Cluster description	*M*	*SD*	*χ*^2^	*p*
Cluster A: Perception of own cognitive and writing ability	4.0	1.2	3.93	.863
Cluster B: Perception of value of feedback	4.1	1.3	3.65	.887
Cluster C: Perception of relationship with tutor	4.1	1.3	4.94	.764
Cluster D: Perception of self-regulation impact	4.0	1.2	7.35	.499

A total of 290 comments to the following two open-ended questions were analyzed to identify the main themes.

#### Q. What did you find to be the most helpful part of the program?

Comments to this question could be classified under 4 major themes: related to personalized instructor support and guidance (62%); related to development of academic skills (29%); related to affective factors (8%); miscellaneous (2%).

The range of comments of what was most helpful to students in RWE indicate the value of the personalized feedback that served students in different ways. Examples of quotes relevant to the question of supporting students in developing their skills as a writer to meet higher education expectations:

*I believe the meetings with my instructor were most helpful because it was not a lesson or lecture, rather it was a* ***conversation about the writing****, the writing style, the content of my journal responses, and my opinions and thoughts about the issues I discussed. This made it* ***fun to be part of this program*** *and was not restricted to forcing myself to read and write on a daily basis.*

(student in group TC)

*I really* ***enjoyed the feedback*** *from my instructor. Also, I found it helpful when I would complete my readings ahead of time since I needed to pick reading material everyday for my RWE journals.*

(student in group TA)

*For me, it is the requirement to work on a daily basis, to post our journals from the readings as it* ***forces the student to read, think, and compose short essays regularly****. The more you do it, the easier it becomes over time*.

(student from group TF)

#### Q. What would you like to see changed in RWE?

Students’ comments for changes clustered around five themes: Related to hiring more instructors, getting more virtual meetings, more/faster instructor feedback and extending the support beyond 1 month (40%); general suggestions for communication about the program (33%); weaknesses related to particular instructor style in some groups (9%); request to be allowed to revise and resubmit some journal entries instead of writing new ones each time (2%). 21% of the comments were actually referring to the program being helpful and which should have been in response to question one such as:

- I feel like it is good as it is from my experience.
- I think everything is perfect right now.
- For now is good.
- I can not think of an improvement right now.
- nothing, i love how the program is.

## Discussion

### Significance of volume of written word output

Academic writing is challenging for students who are unfamiliar with academic writing in Western higher education and have limited Academic English skills. A major barrier for many students is the lack of vocabulary—at least 8000-9000 word families in order to have 98% coverage of the words in an academic text needed for comprehension. A word family is the headword and its various grammatical forms and affixations. For instance, for the word *structure*, the other members of the word family are *structures, structured, unstructured etc.* Vocabulary knowledge has a strong relationship with students’ ability to read and write. Since vocabulary in disciplinary texts are intertwined with disciplinary concepts and terminology, students learning to write about disciplinary topics are basically engaging in practice that enable them to both “write to learn, and learn to write”(Manchon, 2016). Helping students to feel comfortable expressing their thoughts about disciplinary topics expands their active vocabulary acquired through reading disciplinary texts and actively communicating their thoughts.

The volume of writing produced by students at different proficiency levels during the one month in the program differs greatly. As indicated in Fig. 1, students with the lowest Academic proficiency level (Band 1) found it manageable to meet the 250-word goals for writing their daily journal entries and many students voluntarily exceeded that goal. Band 1 students were able to write as much, if not more than their more proficient peers who were in Band 2 and Band 3 suggesting that presenting a practice task as directly relevant to students’ needs (i.e., reading course texts) and then summarizing, paraphrasing as well as evaluatively presenting their inferences or their individual perspective on the material was motivating enough for students to sustain their voluntary engagement. The significance of this observation is that students were effectively acquiring disciplinary vocabulary from reading their course texts (Mežek et al., 2015), while actively using newly acquired vocabulary by writing, expands their repertoire of active vocabulary that can be drawn upon during writing and speaking (Schmitt, 2010).

Writing assignments generally specify the expected length of the assignment. For students who are new to academic writing, reaching that word limit may seem like a daunting task, as expressed by a student in Askham’s (2008) study, “How am I going to find 2500 words to describe my feelings during the workshop…2500 words! That’s called a book isn’t it?” (p. 91). Depending on a student’s Academic English proficiency, the new academic culture where many expectations about academic writing are not explained explicitly to students, but are taken for granted, students with limited English proficiency can find those expectations “inherently threatening, especially through the terrifying scale of assessment tasks” (Askham, 2008, p. 91). In a study of word lengths of 448 written assignments from Year 1 to Year 5 undergraduate programs in one academic year in a Canadian university Graves et al., (2010) found the average word count required in assignments was 1,300 words, and the median was 1000 words while an assignment of more than 12 pages was rare. The average total number of words written voluntarily by students in the RWE program in one month exceeds the word count needed for most assignments. Though an assignment and daily RWE writing are different in nature, the RWE writing that is risk-free serves like the pre-writing and brainstorming students do as they begin to tackle their assignments. For ELLs, the need to write at least 250 words each day is a small step forward. By the end of 28 days, they would have felt more competent with writing in their own words in English, and a 1300 word assignment requirement will not make them panic and resort to AIV. So, this “atomic habit”(Clear, 2018) of writing their original thoughts about their course topic, and the atomic habit of daily thinking more deeply and critically as a result of the responses to their ideas from the instructor add up to a significant capital of disciplinary knowledge and linguistic resources to cope with their course assignments independently.

From the perspective of helping students feel more prepared to write their own assignments instead of seeking inappropriate help or services, setting a daily goal of 250 words to write about disciplinary topics makes it manageable for students to use academic vocabulary and disciplinary terminology to build their comfort level and sense of increasing familiarity and emergent scholarly thinking ability. As illustrated by Fig. 2, extremely low proficiency students (classified as Band 1) likely appreciated this risk-free language usage practice that they wrote as much as students in Band 3 who were more proficient in Academic English. More importantly, giving students the learner agency and opportunity for practice in ways that allow them to incrementally acquire the content knowledge capital, linguistic capital and learn scholarly practices of asserting their own ideas while distinguishing them for source information makes the daily practice motivating and empowers students to uphold academic integrity. As the daily language practice hone students’ efficiency in articulating their thoughts using their expanded vocabulary, low proficiency students kept up with course readings. They were more prepared to independently write their assignments instead of resorting to alternative methods leading to AI violations. Students may resort to cheating services when they “lacked confidence in their ability to complete the assessment” (Yorke et al., 2020, p. 7), as illustrated by a student who said, “if the assignment will cause me to fail it will cost an additional $1500 to repeat the unit compared to only $150 and a small chance of being caught” (Yorke et al., 2020, p. 7). By facilitating students in disciplinary languaging, RWE students’ daily or almost daily dialogic interactions in asynchronous communicaion with their instructor helped them feel comfortable with writing about disciplinary topics, and exercising the agency to incorporate feedback from their instructors to increase their disciplinary and linguistic capital.

### Development of critical thinking, Academic English and writing skills needed at university

For the Yes/No items on the anonymous survey, areas that had been the long-running focus of the program netted 92% and 93% positive responses. However, the newer areas that were emphasized in the re-envisioned program—vocabulary development and in-text citation rated lower. This is likely due to a number of the instructors in the program who had not yet adjusted their teaching style to focus on teaching in-text citations to students in different geographical locations.

The high percentage of positive perception for paraphrasing and summarizing skills (92%); organization of ideas (92%); critical thinking about disciplinary topics (93%); logic and argument structure (92%); awareness of academic audience (84%) likely account for sustaining students’ voluntary investment of time and effort in this reading-into-writing practice.

The low percentage to students’ perception of learning about in-text citation is an indication that more training for instructors drawing on findings from AI research is needed. Students need the experience of purposeful use of citation as a means of evidence-based argument to help them distinguish their thoughts from that of their sources. This need to use citation may be culturally alien to some students. Unlike the expectations to be thoroughly engaged in a culture of enquiry expected in Western academic writing, students from some cultures are used to accept established knowledge and the words of elders. For instance in Szilagyi (2014)'s study, Nigerian students taking an online British university course were shocked to be charged with plagiarism despite feeling a high sense of personal integrity because they were not aware that they need to explicitly cite and reference the work of others.

Since development of authorial voice is necessary in academic writing, the high percentage of Yes responses to summarizing, paraphrasing, organization of ideas and argument structure indicate that this system of supportive training enables students to embark on incrementally strengthening their authorial voice to participate in the academic conversation. The daily practice in developing stronger reader awareness through dialogic interactions with the instructor provide essential practice in communicating on disciplinary topics.

Since Table 4 shows that there is no significant difference across the 9 groups of students supported by different instructors, this approach could be replicable in different teaching contexts once instructors are trained in the basic pedagogy for facilitating learner agency. Learning in this practice-oriented way develops students’ experiential understanding of academic integrity used in connection with acquiring disciplinary knowledge and skills for writing in the disciplines.

### Learner Agency, Self-Regulation and Practicing Academic Integrity

The open-ended responses indicate students’ strong appreciation for communication with the instructor. The nature of the responses from the instructor that are supportive and encouraging guides students in the development of their academic skills, and most importantly the humanistic warm relationship with the student helps the student feel welcomed in the academic community. The sense of belonging likely contributed to students’ voluntarily sustaining their practice in the program. When students feel comfortable and enjoy exchanging ideas and acquiring the notions of academic integrity in the academic environment, they discover agency and ownership and distinguish between their ideas and sources to acquire a scholarly orientation in communicating.

A key strategic element in this program is prioritizing learner agency. Giving learners the agency to choose which of their course texts to read was a strategic component of this approach because of the material’s relevance to the student. In this way the program supported students’ agency and helped them guard against procrastination. When writing about the course topics, students may have more disciplinary knowledge than their RWE instructor putting students in the position of being the expert. Unlike writing an assignment draft or an assignment in a course where the reader is more knowledgeable relative to the student, the relationship between the instructor in the RWE program and the student is more non-hierarchical and the power differential is minimal. This helps students develop greater confidence articulating their thoughts about their course topics.

Learner agency and self-regulation play a major role as students learn the ropes in the academic culture. Learning about Academic Integrity in their Day 2 task raised students’ awareness of the importance of developing their academic literacy and academic writing skills. Practicing source-based disciplinary writing with the instructor as an audience and knowing that the response is safe, supportive and scaffolded helps to make the teaching-learning space one that encourages learner agency.

### Recommendations for Proactive Practice in Academic Integrity

The following recommendations address equity, diversity and inclusion by empowering learners in a proactive manner through a multi-stakeholder approach, and move towards the transformative approach advocated by (Thacker, in press), that is “social, and shaped with and by the students, improving student responsibility for the outcomes of their learning process” (p. 10).

## Recommendation 1: Invest in working *with* learners, giving learners agency to develop competence and confidence in academic writing through risk-free practice

- Work *with* learners as partners in AI Education/Training and give them the agency to focus on what is most relevant to their needs.
- Create opportunities that enable students to share their opinions, engage in exchange of ideas, and develop a good sense of what ideas are theirs and how they distinguish their thoughts from others.
- Make the writing task small enough to be done on a daily basis, and make AI an integral part of the thinking and writing.

### Recommendation 2: Establish proactive start-of-semester learning/practice conditions which are inherently motivating and sustainable

- Set system to make it conducive for equity-deserving students to take on the role of junior-scholar-in-training in sharing with the instructor a new nugget of knowledge from their course readings along with how they make sense of it based on their lived experience, culture, and different ways of knowing.
- Focus on helping students develop fluency when writing in English, and respond to their ideas in ways that engage critical thinking
- Integrate support and course-based needs so tasks required do not contribute to time-burden on their time.

### Recommendation 3: Engage students in developing a scholarly orientation through positive and encouraging interactions and address affective factors for writing/ communication in English

- Develop student confidence and competence through timely supportive feedback.
- Demystify AI in academic writing by having students operate in the system and uphold AI. This way it is not alien to them, and they do not feel like an outsider to the new academic culture. Explicitly teach paraphrasing specific to the student needs encourages improvement of skills and assertion of their opinion to show their perspectives are valued. Students then feel more comfortable articulating their perspectives rather than just borrowing those of others.
- Engage students to draw inferences from what they have read, and to reflect on what they have learned from their own perspectives. This helps students to learn how to engage in scholarly activity.

### Recommendation 4: Provide opportunities for cultural bridging so that students can make the link between their previous educational experience as they learn about expectations in their new environment, for themselves and in communication with others

- Interact with students in a non-hierarchical structure to enable greater intercultural exchanges that help to make the new academic environment and culture more inclusive and less inhibiting for equity-deserving students.
- Reduce the power differential between learner and instructor to encourage students to feel viable in sharing their perspectives that can enrich the teaching-learning dynamics as well as help students feel empowered in their ability to contribute.
- Help students to trust and feel supported so that they realize that the system is not out to catch them on AI, but that the system is *with* them, and to support them.

### Limitations

As this study was done retrospectively and the data was initially collected for program evaluation, we did not have a chance to interview the student participants to gather their perspectives as to what motivated them to sustain their engagement to read and write with academic integrity or what barriers they faced when attempting to do so. Future research could interview students for more in-depth and detailed responses as well as instructors to establish the underlying motivations and dynamics of interaction. Another area of research would be to study to what extent this AI Socialization (which incorporates language development) can help equity-deserving students feel they can develop their authentic identity in writing their ideas related to their course topics without the need to copy or incorporate too much text from source, resulting in AIV.

## Conclusions

This proactive student-driven, instructor facilitated approach that takes an educative emphasis when socializing students to AI, and provides a safe space for ELLs to bring their previous sociocultural, educational and linguistic experiences to their learning about AI using their own course materials resonated with students. This suggest the viability of this approach in creating a developmental space that is not remedial or stigmatized. During the pandemic when remote learning makes students feel lost and isolated while having to cope with a higher volume of written work, this AI Socialization opportunity to engage in a positive experience while mastering both academic language and academic integrity helps students cope with the demands of their course in “digestible” daily participation which resulted in tangible positive outcomes. Students chose to voluntarily write a high volume of journal entries about their course topics which resulted in (a) the opportunity to draw upon previous educational experiences as they made sense of AI in their writing; (b) assuming the junior scholar role daily as they practice articulating their thoughts to their RWE instructor; (c) exponential expansion in vocabulary and academic language constructions that would make it easier for students to write their assignments (d) positive mindset of their own abilities to express their ideas and counter the deficit narrative applied on ELLs and equity-deserving students.

While we make no claim that a one-month support can solve potential plagiarism problems, we argue that a month-long scaffolded learning experience practising academic integrity is a good, productive start for ELLs learning about their course material, and thus making it less likely for them to engage in AIVs. The high volume of writing with supportive responses from the instructor made the almost daily writing routine perceived to be worthwhile by ELLs, and thus they kept up with their practice. This proactive and empowering approach to learning about AI is a powerful way to address individual students’ unique set of learning needs because the students are partners in the process and are actively embodying academic integrity through the AI Socialization process, thus transforming their own learning experience.

## Abbreviations

AI: Academic integrity
AIV: Academic integrity violation
ANOVA: Analysis of Variance
DELNA: Diagnostic English Language Needs Assessment
ELL: English Language Learners
LMS: Learning management system
PELA: Post-entry language assessment
RWE: Reading and Writing Excellence
SES: Socio-economic status
